# Ethnic disparities in metabolic dysfunction-associated steatotic liver disease and clinical outcomes

**DOI:** 10.3389/fendo.2025.1739137

**Published:** 2026-01-09

**Authors:** Yusuke Miyatani, Aoi Ogawa, Tomoki Sempokuya, Chuong Tran, Davis James, Todd Seto, Cecilia Shikuma, Scott K. Kuwada

**Affiliations:** 1 Department of Medicine, John A. Burns School of Medicine, University of Hawai’i, Honolulu, HI, United States; 2 Department of Medicine, The Queen’s Medical Center, Honolulu, HI, United States; 3 University of Hawaii Cancer Center, Honolulu, HI, United States

**Keywords:** Asian, clinical outcome, ethnic disparity, metabolic dysfunction-associated steatotic liver disease, mortality, Native Hawaiian and Pacific Islanders

## Abstract

**Background:**

While metabolic dysfunction-associated steatotic liver disease (MASLD) has become increasingly prevalent worldwide, multi-ethnic differences within a shared geography and lifestyle, have not been fully examined. What remains poorly characterized are the clinical outcomes for ethnic minorities in the U.S., particularly Asians and Native Hawaiian and Pacific Islanders (NHPI), compared with White people/persons/person.

**Methods:**

Adults (aged ≥18 years) diagnosed with MASLD between January 2008 and December 2018 were identified in the TriNetX national database using the ICD-10 codes and followed outcomes through August 2025. Propensity score matching was conducted to compare Asians and NHPI with White people/persons/person with MASLD, adjusting for age, gender, body mass index, hypertension, type 2 diabetes, hyperlipidemia, and smoking status. Hazard ratios (HR) with 95% confidence intervals were estimated for the major outcomes: all-cause mortality, cirrhosis, and hepatocellular carcinoma (HCC). The effects of extrahepatic diseases related to metabolic diseases, such as myocardial infarction (MI), heart failure (HF), chronic kidney disease (CKD), and non-HCC cancer, were examined in the analyses.

**Results:**

A total of 188,328 White, 14,475 Asian, and 2,390 NHPI patients living in the U.S. (United States of America) with MASLD were identified with a median follow-up of over 8 years. After propensity score matching, Asian and NHPI demonstrated significantly lower rates of cirrhosis than White people/persons/person. In Asians, the risk of HCC increased with longer follow-up period, and with ≥5 years of follow-up, HCC risk significantly exceeded that of White people/persons/person. Asians had lower rates of CKD, MI, HF, and non-HCC cancers than White people/persons/person. NHPI had a significantly lower rate of non-HCC cancers but a higher risk of CKD compared with White people/persons/person. All-cause mortality was lower among Asians, but not in NHPI, compared with White people/persons/person.

**Conclusions:**

In a large, multiethnic U.S. cohort of MASLD, Asians and NHPI showed distinct outcome profiles. Ethnicity-tailored MASLD management strategies should be further explored.

## 1 Introduction

Metabolic dysfunction-associated steatotic liver disease (MASLD), the updated classification that includes patients formerly diagnosed with non-alcoholic fatty liver disease (NAFLD), currently affects over 30% of the general population worldwide (1, 2). In MASLD, persistent hepatic inflammation promotes oxidative stress and fibrogenesis, contributing not only to major adverse liver outcomes such as cirrhosis and hepatocellular carcinoma (HCC) (3) but also to extrahepatic events, including cardiovascular disease, chronic kidney disease (CKD), and extrahepatic cancers, which together contribute substantially to mortality (4–7). As the incidence of MASLD continues to rise in parallel with the global epidemics of obesity and type 2 diabetes, two principal drivers of MASLD (2), identifying populations at greatest risk for these complications has become a public health priority (8).

As the progression of MASLD is closely related to lifestyle, environmental, and genetic factors, the disease burden is unevenly distributed across ethnic groups (9, 10). In the United States (U.S.), Hispanics have the highest prevalence of MASLD and metabolic dysfunction-associated steatohepatitis (MASH), driven in part by a higher population frequency of the *PNPLA3* I148M variant, while African Americans have lower rates compared to White people/persons/person (11) (12). By contrast, Asian and other ethnic minorities, which collectively comprise approximately 10% of the U.S. population, have not been as well studied across the U.S. despite a substantial MASLD burden in these populations (13, 14). In particular, evidence regarding MASLD among Native Hawaiian and Pacific Islanders (NHPIs), a group with high rates of obesity and type 2 diabetes, is scarce, because national datasets often aggregate NHPIs with Asians or exclude them entirely (14–16). As lifestyle and environmental factors are strongly shaped by culture and geography, clarifying the magnitude and nature of ethnic disparities in these underrepresented populations living in the U.S. and sharing a common Western lifestyle is essential to inform screening strategies, clinical interventions, and the design of future clinical trials in MASLD.

In this study, we aimed to characterize MASLD and evaluate clinical outcomes among ethnic minorities in the U.S., focusing on Asians and NHPIs compared with White people/persons/person, with particular attention to cirrhosis, HCC, and all-cause mortality. In secondary analyses, we also examined extrahepatic complications, including cardiovascular and renal events, and extrahepatic malignancies.

## 2 Materials and methods

We conducted a retrospective cohort study using the TriNetX US Network to investigate ethnic variation in MASLD as reflected in clinical outcomes, including mortality, cirrhosis, and HCC. The TriNetX US network integrates de-identified data from over 130 million patients across more than 70 healthcare organizations (HCOs) throughout the U.S. All data used in this analysis were de-identified in compliance with the Health Insurance Portability and Accountability Act (HIPAA) Privacy Rule, with only aggregate counts and statistical summaries accessible for analysis. Analysis was completed on August 29, 2025.

### 2.1 Study participants and cohorts

#### 2.1.1 Data source and patient identification

This study utilized the TriNetX US Network, which integrates de-identified data and comprised 131,882,080 electronic medical records (EMRs) at the time of data extraction. Patient identification within the TriNetX database was based on the presence of corresponding International Classification of Diseases, Tenth Revision (ICD-10) codes in their electronic health records (EHRs). Adult patients (aged ≥18 years) newly diagnosed with MASLD between January 2008 and December 2018 were identified. We extracted clinical data for patients meeting the criteria for MASLD, defined as having an ICD-10 code for K76.0 (Fatty [change of] liver, not elsewhere classified) or K75.81 (Nonalcoholic steatohepatitis) along with at least one of the following metabolic risk factors: Body Mass Index (BMI) ≥25 kg/m2, hypertension, type 2 diabetes, or hyperlipidemia. For Asian patients, the BMI threshold for diagnosis was adjusted to ≥23 kg/m2 to account for the lower average BMI observed in Asian populations with MASLD (17). Because our study period predates the introduction of the MASLD nomenclature, we identified cases using Non-Alcoholic Fatty Liver Disease (NAFLD)-related ICD-10 codes (K76.0 and K75.81) in combination with metabolic risk factors (18). Given the strong concordance between the previously utilized criteria for NAFLD and the current diagnostic criteria for MASLD (19), we retrospectively harmonized these diagnoses to MASLD and use MASLD terminology throughout this manuscript. Self-reported patient ethnicity information was obtained directly from the TriNetX database.

#### 2.1.2 Exclusion criteria

Patients with co-existing hepatitis B or C, alcohol-related liver disease, or other chronic liver diseases (autoimmune hepatitis, hemochromatosis, alpha 1 anti-trypsin deficiency, Wilson’s disease) were excluded. Patients who developed any outcome before the index date, defined as the date of MASLD diagnosis, were also excluded from outcome analyses. To mitigate detection bias for clinical outcomes (excluding all-cause mortality analysis), we included only patients with a minimum follow-up period of one year following the MASLD diagnosis. All patients meeting the inclusion criteria were included in the all-cause mortality analysis, regardless of the length of follow-up, as mortality events are comprehensively captured in the source EHRs. For ethnicity, Hispanics were excluded from White people/persons/person. Detailed selection and exclusion criteria are outlined in **Supplementary Tables 1 and 2.**

### 2.2 Outcome measures

**Figure 1:**
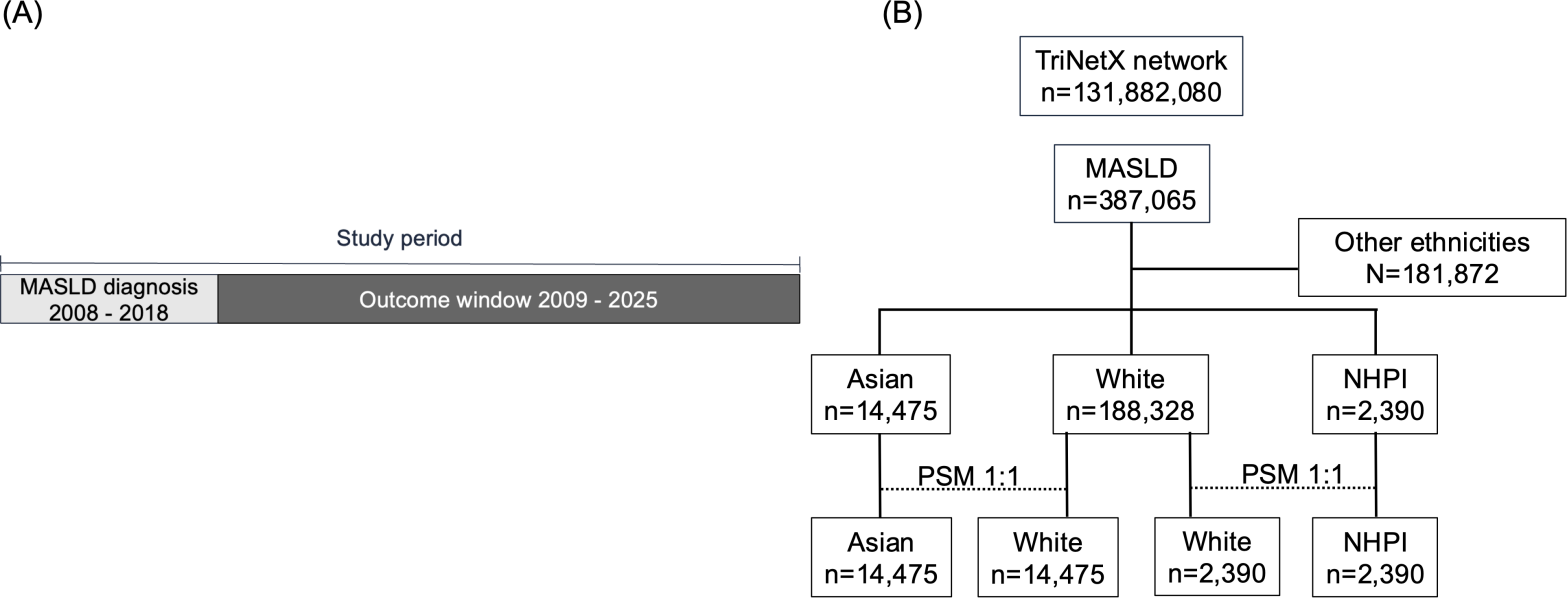
Study overview **(A)** A timeline illustrating the study period from participant inclusion to the end of data collection. **(B)** Study flow diagram. Abbreviations: MASLD, metabolic dysfunction–associated steatotic liver disease; NHPI, Native Hawaiian/Pacific Islander; PSM, propensity score matching.

The major outcomes for MASLD included cirrhosis, HCC, and all-cause mortality. All-cause mortality was precisely defined by the presence of the term “deceased” in the EMR or the assignment of ICD-10 code R99. Secondary outcomes included specific cardiovascular events (myocardial infarction, heart failure), renal outcomes (CKD stage ≥3), and a panel of non-HCC malignancies, specifically: colon, rectosigmoid, rectum, prostate, uterus, breast, lung, stomach, and melanoma cancers. Follow-up for outcomes analysis concluded in September 2025 (**Figure 1A**). ICD-10 codes were utilized for defining clinical outcomes delineated in **Supplementary Table 3**.

### 2.3 Statistical analysis

#### 2.3.1 Baseline characteristics and comparisons

Baseline characteristics of the cohort were summarized using means and standard deviations for continuous variables and percentages for categorical variables. We compared baseline characteristics, including age, BMI, type 2 diabetes, hypertension, hyperlipidemia, and smoking history, across different ethnic groups. Student’s t-test was used for continuous variables, and the Chi-squared test was used for categorical variables, as appropriate. Two-sided p-values were calculated, and a significance level of α<0.05 was considered statistically significant.

#### 2.3.2 Propensity score matching

A 1:1 propensity score matching (PSM) analysis was conducted to balance baseline covariates and assess clinical outcomes. Matching factors included age, gender, BMI, hypertension, type 2 diabetes, hyperlipidemia, and smoking status. To accurately capture tobacco use for PSM, both nicotine dependence (ICD-10 F17) and tobacco use (ICD-10 Z72.0) codes were utilized. For all-cause mortality analysis, we additionally adjusted for baseline statin and/or aspirin use at the MASLD diagnosis, given prior evidence associating their use with mortality (20, 21) (**Supplementary Table 4**). Propensity scores were estimated using logistic regression, implemented via the LogisticRegression function from the scikit-learn package in Python. Matching was performed using nearest-neighbor algorithms with a caliper width of 0.1 on the pooled standardized differences of the propensity scores.

#### 2.3.3 Outcome analysis

For non-mortality outcomes (cirrhosis, HCC, secondary outcomes), we fit Cox proportional-hazards models and reported the Hazard Ratio (HR) with 95% confidence intervals (CIs) to quantify differences by ethnicity. White people/persons/person were used as the reference group for comparisons with Asians and NHPI. In prespecified sub-analyses, we repeated the models in cohorts restricted to patients with at least 3 and 5 years of follow-up period to assess time-dependent effects since the MASLD diagnosis. The proportional hazards assumption was evaluated using the test based on scaled Schoenfeld residuals implemented in the TriNetX analytics platform, and no major violations of the proportional hazards assumption were detected for the ethnicity covariates in the primary Cox models. Survival analysis for all-cause mortality was performed using the Kaplan-Meier method. The inter-group difference was analyzed with a log-rank test. All statistical analyses were conducted on the TriNetX Live research platform (TriNetX LLC, Cambridge, MA, USA).

## 3 Results

### 3.1 Demographic characteristics

A total of 188,328 White, 14,475 Asian, and 2,390 NHPI patients with MASLD were identified with a median follow-up of 8.4 years, 8.1 years, and 8.5 years, respectively. After 1:1 propensity score matching, 14,475 Asian and 2,390 NHPI were matched to an equal number of White people/persons/person. (**Figure 1B**) Among the major significant differences found in the baseline cohort, Asians had the lowest mean BMI (29.0) (**Table 1A**), while NHPIs had the youngest mean age of diagnosis of MASLD (49.8 years), highest mean BMI (36.9), and highest rates of type 2 diabetes (38.3%), hypertension (59.6%), and nicotine dependence (15.8%) (**Table 1B**). The demographic characteristics of mortality and sub-analyses were described in **Supplementary Tables 5-7**.

**Table 1:** Baseline characteristics of patients with MASLD before and after propensity-score matching.

(A) White versus Asian with MASLD						
Covariate	Pre-propensity score matching		p-value	Post-propensity score matching		p-value
	White	Asian		White	Asian	
Sample size	188,328	14,475		14,475	14,475	
Age at index event - mean ± SD (years)	54.6 ± 13.5	53.8 ± 13.8	<0.001	53.8 ± 13.9	53.8 ± 13.8	0.900
Sex (%)						
Female	56.2	55.3	0.032	55.2	55.3	0.934
Male	43.8	44.7	0.029	44.8	44.7	0.962
Type 2 Diabetes Mellitus	54,545 (29.0%)	4,509 (31.2 %)	< 0.001	4,541 (31.4 %)	4,506 (31.2 %)	0.685
Hyperlipidemia	73,599 (39.1 %)	6,085 (42.0 %)	< 0.001	6,185 (42.7 %)	6,085 (42.0 %)	0.234
Hypertension	98,501 (52.3 %)	7,474 (51.6 %)	0.12	7,586 (52.4%)	7,474 (51.6 %)	0.188
BMI - mean ± SD (kg/m^2)	34.9 ± 7.6	29.0 ± 5.4	< 0.001	29.6 ± 5.8	29.0 ± 5.4	<0.001
BMI group (%)						
< 25 kg/m^2	9.3	19.6	< 0.001	18.6	19.6	0.030
25–30 kg/m^2	27.1	33.1	< 0.001	34.4	33.1	0.025
30–40 kg/m^2	46.7	22.5	< 0.001	22.7	22.5	0.683
40–50 kg/m^2	18.2	2.9	< 0.001	2.9	3.0	0.703
50 < kg/m^2	5.1	0.7	< 0.001	0.6	0.7	0.185
Nicotine dependence	21,020 (11.2%)	802 (5.5 %)	< 0.001	762 (5.3 %)	802 (5.5 %)	0.298
Tobacco use	4,908 (2.6 %)	163 (1.1 %)	< 0.001	106 (0.7 %)	163 (1.1 %)	<0.001
(B) White versus NHPI with MASLD						
Covariate	Pre-propensity score matching		p-value	Post-propensity score matching		p-value
	White	NHPI		White	NHPI	
Sample size	188,328	2,390		2,390	2,390	
Age at index event - mean ± SD (years)	54.6 ± 13.5	49.8 ± 13.5	< 0.001	49.8 ± 13.5	49.8 ± 13.5	0.914
Sex (%)						
Female	56.2	59.9	< 0.001	60.1	59.9	0.906
Male	43.8	40.1	< 0.001	39.9	40.1	0.906
Type 2 Diabetes Mellitus	54,545 (29.0%)	916 (38.3 %)	< 0.001	905 (37.9%)	916 (38.3 %)	0.743
Hyperlipidemia	73,599 (39.1 %)	1,052 (44.0 %)	< 0.001	1,069 (44.7 %)	1,052 (44.0 %)	0.621
Hypertension	98,501 (52.3 %)	1,424 (59.6 %)	< 0.001	1,433 (60.0 %)	1,424 (59.6 %)	0.791
BMI - mean ± SD (kg/m^2)	34.9 ± 7.6	36.9 ± 8.8	< 0.001	36.8 ± 8.7	36.9 ± 8.8	0.748
BMI group (%)						
< 25 kg/m^2	9.3	5.4	< 0.001	5.5	5.4	0.799
25–30 kg/m^2	27.1	14.5	< 0.001	14.4	14.5	0.967
30–40 kg/m^2	46.7	30.3	< 0.001	30	30.3	0.85
40–50 kg/m^2	18.2	17.2	0.235	16.6	17.2	0.537
50 < kg/m^2	5.1	6.3	0.007	6.1	6.3	0.764
Nicotine dependence	21,020 (11.2 %)	378 (15.8 %)	< 0.001	352 (14.7 %)	378 (15.8 %)	0.296
Tobacco use	4,908 (2.6 %)	100 (4.2 %)	< 0.001	82 (3.4 %)	100 (4.2 %)	0.174

(A) White versus Asian patients; (B) White versus NHPI patients. MASLD, metabolic dysfunction–associated steatotic liver disease; NHPI, Native Hawaiian/Pacific Islander.

### 3.2 Clinical outcomes

**Figure 2:**
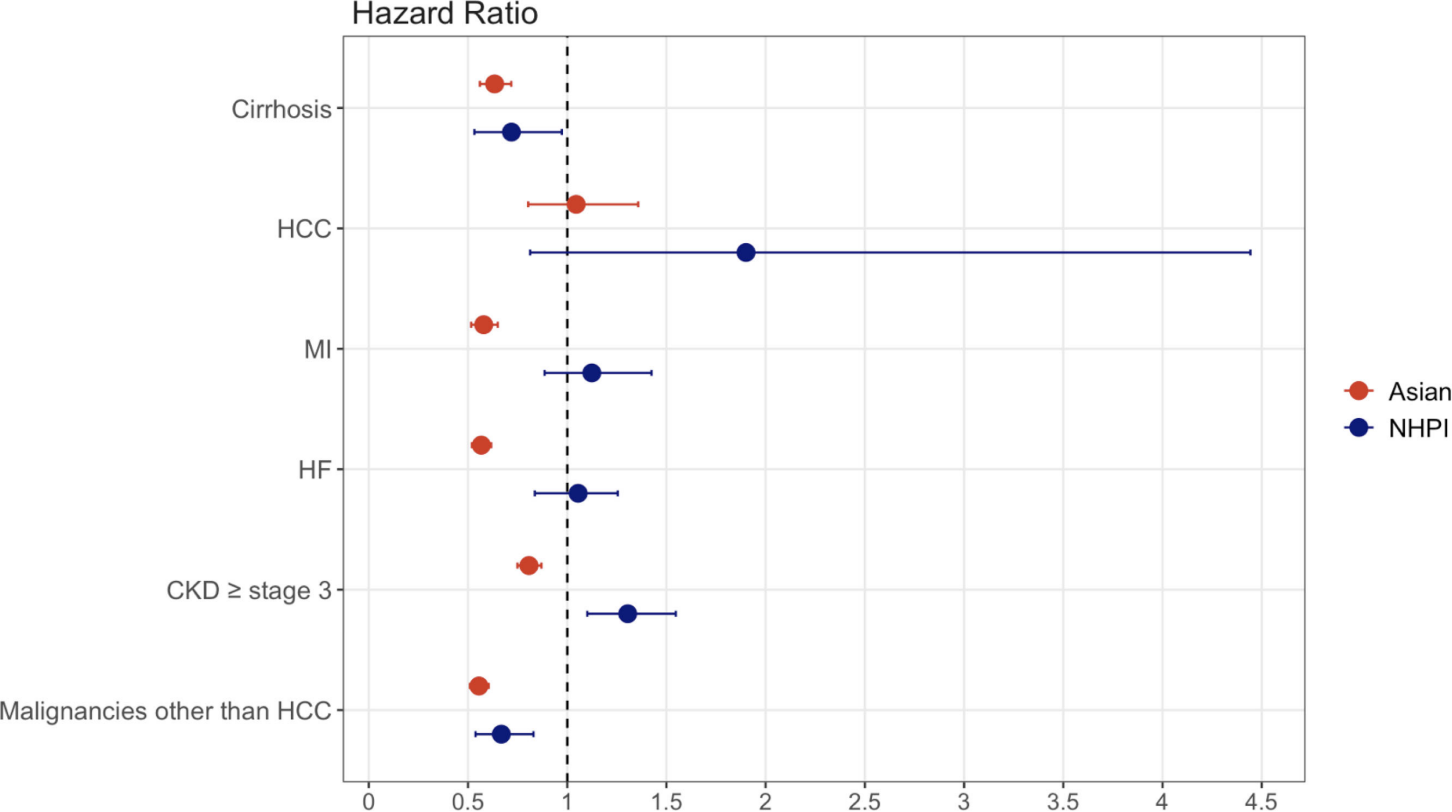
Hazard ratios and 95% confidence intervals (CI) for clinical outcomes during the follow-up period since the MASLD diagnosis. See also **Supplementary Figure 1** for time-dependent hazard ratios. Asian and NHPI were compared with White after propensity score matching using age, gender, BMI, hypertension, diabetes, hyperlipidemia, and smoking status. Error bars represent 95% CI. MASLD, metabolic dysfunction–associated steatotic liver disease; HCC, hepatocellular carcinoma; MI, myocardial infarction; HF, heart failure; CKD, chronic kidney disease; NHPI, Native Hawaiian/Pacific Islander.

*Major liver outcomes* - Both Asian and NHPI demonstrated significantly lower risks of cirrhosis compared with White people/persons/person (Asians: HR 0.63, 95% CI 0.56-0.72; NHPI: HR 0.72, 95% CI 0.53-0.93). By contrast, the risk of HCC did not differ significantly (Asians: HR 1.05, 95% CI 0.80-1.36; NHPI: HR 1.90, 95% CI 0.81-4.44) (**Figure 2**). However, in the sub-analysis restricted to patients with ≥5 years of follow-up, Asians showed a significantly higher rate of HCC compared with White people/persons/person (**Supplementary Figure 1**). Of note, the risk of HCC increased in both Asian and NHPI in a time-dependent fashion: Asians with ≥3 years follow-up: HR 1.16, 95% CI 0.87-1.53; Asians with ≥5 years follow-up: HR 1.39, 95% CI 1.01-1.89; NHPI with ≥3 years follow-up: HR 1.68 95% CI 0.74-3.81; NHPI with ≥5 years follow-up: HR 2.54, 95% CI 0.99-6.56).

*Extrahepatic outcomes* - Compared to White people/persons/person, Asians had lower risks of CKD (HR 0.81, 95% CI 0.75-0.87), MI (HR 0.58, 95% CI 0.52-0.65), HF (HR 0.57, 95% CI 0.52-0.62), and cancers other than HCC (HR 0.56, 95% CI 0.51-0.60). Among NHPI, the risk of cancers other than HCC was lower than in White people/persons/person (HR 0.67, 95% CI 0.54-0.83), whereas the risk of CKD was higher (HR 1.31, 95% CI 1.10-1.55) (**Figure 2**).

### 3.3 All-cause mortality

**Figure 3:**
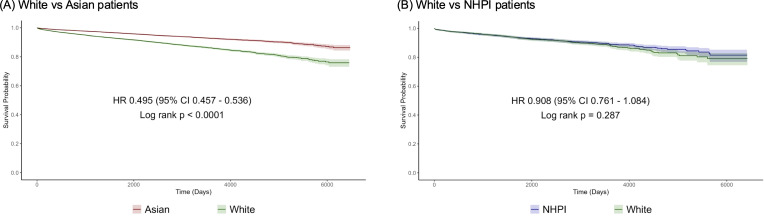
Kaplan-Meier curves for all-cause mortality since the date of MASLD diagnosis. **(A)** White versus Asian patients; **(B)** White versus NHPI patients. MASLD, metabolic dysfunction–associated steatotic liver disease; NHPI, Native Hawaiian/Pacific Islander; HR, hazard ratio; CI, confidence interval.

Kaplan-Meier curves for all-cause mortality are shown in **Figure 3**. The median follow-up of White, Asian, and NHPI was 7.9 years, 7.5 years, and 8.1 years, respectively. Asian patients with MASLD showed a significantly lower risk for all-cause mortality than White patients (HR 0.50, 95% CI 0.46–0.54). In contrast, NHPI patients did not differ significantly compared with White people/persons/person (HR 0.91, 95% CI 0.76–1.08).

## 4 Discussion

Ethnic differences in baseline characteristics in MASLD carry practical implications for healthcare. In our study, Asians with MASLD showed the lowest BMI compared to White people/persons/person and NHPI, consistent with previous reports (14, 22). Based on anthropometric studies, the definition of lean in Asians consists of a lower threshold BMI than non-Asians, where lean is defined as lower than 23 kg/m^2^, overweight as BMI of 23.0-27.5 kg/m^2^, while in non-Asians lean is defined as BMI less than 25 kg/m^2^ and overweight is defined as BMI of 25-29.9 kg/m^2^ (17). A meta-analysis using a global population showed approximately 40% of MASLD patients were categorized as non-obese and 20% as lean (17). In that study, lean and non-obese groups developed hepatic and extrahepatic comorbidities overall, regardless of lower BMI. In addition to BMI, body fat composition is also known to differ among races. Lim et al. reported Asian-American women with hyperlipidemia had greater abdominal and visceral fat compared to White women with similar BMIs (23). The ethnic differences in fat distribution and BMI have important implications in the context of evaluating and treating patients for MASLD. Our study showed that the risks of applying a BMI threshold for obesity in White people/persons/person to Asians could result in missing Asians with MASLD.

With regards to the major outcomes of all-cause mortality and cirrhosis being significantly lower in Asians than in White people/persons/person with MASLD, it is possible that the lower mean BMI in Asians may be an important factor. Further studies will be needed to determine if the differential types of fatty deposition in White people/persons/person and Asians can influence the outcomes of MASLD. NHPI had significantly lower rates of cirrhosis than White people/persons/person but had significantly higher average BMIs.

NHPIs had the youngest age of diagnosis of MASLD with the highest number of metabolic factors, including BMI, type 2 diabetes, hypertension, and tobacco smoking. A large population-based cohort in the U.S. showed that NHPIs have the highest prevalence of obesity and type 2 diabetes in the general population (24). Interestingly, NHPI with MASLD were significantly younger compared with Asians and White people/persons/person, implying that earlier screening and clinical intervention for MASLD may be important for preventing complications in NHPI.

Our study showed Asians demonstrated a significantly lower risk of cirrhosis compared to White people/persons/person after propensity score matching. Lower progression risk of MASLD to cirrhosis in Asians is consistent with the previous longitudinal study using a large MASLD cohort in the U.S (6). The study included more than two thousand Asian-Americans who had a significantly lower risk of the 5-year incidence of cirrhosis after multivariable adjustment. On the other hand, Mohanty et al. conducted a single-center study using histology-confirmed MASLD comparing different races and found that Asians had higher grades of hepatocyte ballooning than White people/persons/person, but the association with clinical outcomes was not assessed (25). While ballooning hepatocyte degeneration is one of the key histologic features of MASH associated with liver-related outcomes in general, further studies to investigate the association between histology and clinical outcomes among different ethnic groups are needed (26). Likewise, NHPI with MASLD had a lower risk of developing cirrhosis than White people/persons/person in our study. It is important to note that many Native Hawaiians in the U.S. have Asian ancestry (27). No direct comparisons of cirrhosis-risk between NHPI and White people/persons/person with MASLD are available, as current studies aggregated Asian and NHPI. A possible explanation for a lower risk of cirrhosis in Asians is the different genetic susceptibility to MASLD progression among ethnic groups. Wang et al. found that NHPI had a lower rate of the *PNPLA3* allele compared with Hispanics and White people/persons/person, which is known to influence disease susceptibility and progression of liver disease in MASLD (28). Genetic alterations in the ALDH2 gene that are very common in East-Asians have been associated with worse outcomes in MASLD in those who consume ethanol regularly (29). In addition, many Native Hawaiians share Asian ancestry, which may influence their clinical course similarly to Asians (27). Even though our study was performed in the U.S., we cannot discount environmental factors unique to NHPI communities, such as diet composition and patterns of physical activity, that could modulate liver disease outcomes.

Of note, in our sub-analyses stratified by minimum follow-up duration, Asians demonstrated a higher risk of developing HCC compared with White people/persons/person in a time-dependent manner, reaching statistical significance only among those with ≥ 5 years of follow-up, despite the lower risk of cirrhosis in Asians than White people/persons/person. NHPI showed a similar trend. One plausible explanation is chronic hepatitis B, which could not be excluded by serological tests alone. Chan et al. showed that positive hepatitis B core (HBc) antibody in those with prior exposure to HBV, not current infection, was associated with HCC in MASLD in a multicenter Asian study (30). HBc antibody positivity due to maternal-fetal HBV transmission is still more common in recent Asian immigrants and Pacific Islanders living in the U.S. and is an established HCC risk factor in steatotic liver disease (31, 32). Previous studies have shown that HCC in Asians may arise in patients with chronic HBV-induced hepatitis and prior to the development of cirrhosis (33). Data from a multicenter study in France demonstrated up to one-third of MASLD-related HCC developed in the absence of cirrhosis, supporting an alternative pathway without cirrhosis but through lipotoxic or inflammatory mechanisms (34). These data underscore the potential need for molecular genetic studies for the detection of HBV in Asians and NHPI with MASLD, especially when positive for HBc antibody.

For extrahepatic outcomes, Asians with MASLD demonstrated significantly lower risk in all evaluated outcomes, including cardiovascular events, CKD, and non-hepatic cancers, than White people/persons/person after propensity-score matching, consistent with prior studies (6, 35, 36). While direct, head-to-head evidence on CKD risk between the two groups within MASLD is limited, the observed difference may reflect a greater microvascular risk burden among White people/persons/person (37). Importantly, these findings do not obviate the need for intensified screening for these diseases and possible primary or secondary intervention in Asians, given that patients with MASLD have higher risks of these extrahepatic complications than the general population, which was not addressed in our study (7). While there is no direct comparison regarding CKD risks between NHPI and White people/persons/person with MASLD, a significantly higher risk of CKD in NHPI in our cohort is consistent with U.S. national data showing the highest rate of end-stage renal disease in NHPI, largely attributable to diabetic nephropathy (38).

Asians demonstrated a significantly lower risk of all-cause mortality compared with White people/persons/person after propensity score-matched analysis, consistent with a previous study (6). While the direct cause of mortality was not possible to discern in our study, it is possible that lower rates of intra- and extrahepatic complications in MASLD contributed to their better prognosis. VoPham et al. studied US veteran data and found that non-Hispanic White people/persons/person with cirrhosis due to any etiology showed the highest all-cause mortality compared to other ethnic groups, including Asian and NHPI (39). However, a subgroup analysis focusing on MASLD cirrhosis did not show a significant difference between White people/persons/person and Asians/NHPI, suggesting non-hepatic causes of mortality may be responsible for the difference. A nationwide Swedish cohort revealed that patients with MASLD showed a nearly doubled all-cause mortality rate compared to the general population (4). In that study, those with extrahepatic cancer and cardiovascular disease in MASLD had the highest cumulative incidence of death within 15 years. In our study, it should be noted that there was no significant difference in all-cause mortality between NHPI and White people/persons/person with MASLD, regardless of the lower rate of extrahepatic cancer in NHPI. This could suggest mortality in NHPI was more influenced by cardiovascular events, CKD, and other unmeasured conditions, given their higher rate of baseline metabolic diseases. Further study is needed to elucidate the cause of mortality based on different ethnic groups.

Our findings have important implications for ethnicity-specific MASLD management. For Asians, lower BMI thresholds should be used to trigger MASLD screening in clinical practice, consistent with the current consensus that overweight and metabolic risk occur at lower BMI values in this population (40). In addition, given the potential for a higher long-term risk of HCC despite a lower risk of cirrhosis, HCC surveillance may warrant particular consideration in Asians with long-standing MASLD, especially those positive for HBc antibody. For NHPI, the younger age at MASLD diagnosis and the higher prevalence of multiple metabolic risk factors (type 2 diabetes, hypertension, hyperlipidemia, higher BMI, and smoking) suggest a need for earlier and more intensive screening for both MASLD and cardiometabolic comorbidities.

Our study includes several limitations. We were unable to assess the severity of liver fibrosis using histology in the TriNetX database. Misclassification bias, mainly false negative results, could not be avoided given the reliance of the TriNetX database on ICD-10 codes. We were unable to adjust for newer medications such as glucagon-like peptide-1 agonists and sodium-glucose cotransporter 2 inhibitors because their FDA approvals overlapped minimally with our enrollment window. Sampling bias from the comparison with different matched references due to the large imbalance in sample size could not be excluded, as TriNetX only allows binary propensity score matching. Ethnicity in our study was self-reported. Some residual imbalance persisted between White people/persons/person and Asians after propensity matching, particularly for BMI and smoking (**Table 1A**). For example, mean BMI remained slightly higher among White people/persons/person than Asians (29.6 vs 29.0 kg/m^2^; p < 0.001). This pattern is consistent with the underlying BMI distribution and our use of a lower BMI threshold for MASLD inclusion in Asians (BMI ≥23 kg/m2 rather than ≥25 kg/m^2^ in White people/persons/person and NHPI), reflecting the consensus that Asians should have a lower cut-off for being overweight (17, 41). Given the large sample size, such small absolute differences are expected to yield statistically significant p-values. For smoking, Asians had a slightly higher prevalence than White people/persons/person after propensity matching, whereas White people/persons/person had a significantly higher smoking prevalence before matching. Importantly, these patterns of residual imbalance would be expected to attenuate rather than exaggerate the observed differences in clinical outcomes. After matching, White people/persons/person, who had worse outcomes overall, constituted a healthier subset with substantially lower BMI and lower smoking prevalence than the original White cohort, whereas BMI in Asians changed little and smoking prevalence became slightly higher than in White people/persons/person. Thus, our findings are unlikely to be explained solely by incomplete adjustment and may in fact underestimate the true differences in clinical outcomes between groups. Our study lacked data on genetic variants such as those in the *PNPLA3* and *ADH2* genes that may influence different outcomes in MASLD. Lastly, the data relating to confounding factors such as environmental exposure, diet, physical activity, socioeconomic disparities, and access to health care were not available in this database.

Our study, however, has several strengths. First, there is a good representation of patients of differing ethnicities who share a Western society and environment. Second, the follow-up period affords sufficient person-time to capture clinically meaningful events. Finally, we included an unprecedented number of NHPI, a group often excluded or aggregated with Asians in prior research.

In conclusion, our findings highlight the importance of ethnic disparities in the management of MASLD.

## Data Availability

The original contributions presented in the study are included in the article/**Supplementary Material**. Further inquiries can be directed to the corresponding author.

## References

[1] Rinella ME Lazarus JV Ratziu V Francque SM Sanyal AJ Kanwal F . A multisociety Delphi consensus statement on new fatty liver disease nomenclature. J Hepatol. (2023) 79:1542–56. doi: 10.1016/j.jhep.2023.06.003, PMID: 37364790

[2] Wong VW Ekstedt M Wong GL Hagström H . Changing epidemiology, global trends and implications for outcomes of NAFLD. J Hepatol. (2023) 79:842–52. doi: 10.1016/j.jhep.2023.04.036, PMID: 37169151

[3] Hagström H Shang Y Hegmar H Nasr P . Natural history and progression of metabolic dysfunction-associated steatotic liver disease. Lancet Gastroenterol Hepatol. (2024) 9:944–56. doi: 10.1016/S2468-1253(24)00193-6, PMID: 39243773

[4] Issa G Shang Y Strandberg R Hagström H Wester A . Cause-specific mortality in 13,099 patients with metabolic dysfunction-associated steatotic liver disease in Sweden. J Hepatol. (2025) 83(3):643–51. doi: 10.1016/j.jhep.2025.03.001, PMID: 40139508

[5] Simon TG Roelstraete B Khalili H Hagström H Ludvigsson JF . Mortality in biopsy-confirmed nonalcoholic fatty liver disease: results from a nationwide cohort. Gut. (2021) 70:1375–82. doi: 10.1136/gutjnl-2020-322786, PMID: 33037056 PMC8185553

[6] Nguyen VH Le I Ha A Le RH Rouillard NA Fong A . Differences in liver and mortality outcomes of non-alcoholic fatty liver disease by race and ethnicity: A longitudinal real-world study. Clin Mol Hepatol. (2023) 29:1002–12. doi: 10.3350/cmh.2023.0205, PMID: 37691484 PMC10577349

[7] Targher G Byrne CD Tilg H . MASLD: a systemic metabolic disorder with cardiovascular and Malignant complications. Gut. (2024) 73:691–702. doi: 10.1136/gutjnl-2023-330595, PMID: 38228377

[8] Vitale A Svegliati-Baroni G Ortolani A Cucco M Dalla Riva GV Giannini EG . Epidemiological trends and trajectories of MAFLD-associated hepatocellular carcinoma 2002-2033: the ITA. LI.CA database. Gut. (2023) 72:141–52. doi: 10.1136/gutjnl-2021-324915, PMID: 34933916

[9] Sveinbjornsson G Ulfarsson MO Thorolfsdottir RB Jonsson BA Einarsson E Gunnlaugsson G . Multiomics study of nonalcoholic fatty liver disease. Nat Genet. (2022) 54:1652–63. doi: 10.1038/s41588-022-01199-5, PMID: 36280732 PMC9649432

[10] Ochoa-Allemant P Marrero JA Serper M . Racial and ethnic differences and the role of unfavorable social determinants of health across steatotic liver disease subtypes in the United States. Hepatol Commun. (2023) 7(12):e0324. doi: 10.1097/HC9.0000000000000324, PMID: 38051551 PMC10697602

[11] Tesfai K Pace J El-Newihi N Martinez ME Tincopa M Loomba R . Disparities for hispanic adults with metabolic dysfunction-associated steatotic liver disease in the United States: A systematic review and meta-analysis. Clin Gastroenterol Hepatol. (2024) 23(2):236–49. doi: 10.1016/j.cgh.2024.06.038, PMID: 39025254

[12] Rich NE Oji S Mufti AR Browning JD Parikh ND Odewole M . Racial and ethnic disparities in nonalcoholic fatty liver disease prevalence, severity, and outcomes in the United States: A systematic review and meta-analysis. Clin Gastroenterol Hepatol. (2018) 16:198–210.e2. doi: 10.1016/j.cgh.2017.09.041, PMID: 28970148 PMC5794571

[13] Truong E Yeo YH Cook-Wiens G Muthiah M Yang JD Sundaram V . Nonalcoholic fatty liver disease prevalence and severity in Asian Americans from the national health and nutrition examination surveys 2017-2018. Hepatol Commun. (2022) 6:2253–61. doi: 10.1002/hep4.1981, PMID: 35527706 PMC9426392

[14] Parsa AA Azama KA Vawer M Ona MA Seto TB . Prevalence study of MASLD in adolescent and young adult pacific islanders and asians living in hawai’i. J Endocr Soc. (2024) 8:bvad165. doi: 10.1210/jendso/bvad165, PMID: 38249431 PMC10797323

[15] Mau MK Sinclair K Saito EP Baumhofer KN Kaholokula JK . Cardiometabolic health disparities in native Hawaiians and other Pacific Islanders. Epidemiol Rev. (2009) 31:113–29. doi: 10.1093/ajerev/mxp004, PMID: 19531765 PMC2893232

[16] Taira DA Ranken MS Seto BK Davis J Hermosura AH Porter C . Representation of native hawaiian and pacific islander individuals in clinical trials. JAMA Netw Open. (2024) 7:e2442204. doi: 10.1001/jamanetworkopen.2024.42204, PMID: 39470635 PMC11522938

[17] Ye Q Zou B Yeo YH Li J Huang DQ Wu Y . Global prevalence, incidence, and outcomes of non-obese or lean non-alcoholic fatty liver disease: a systematic review and meta-analysis. Lancet Gastroenterol Hepatol. (2020) 5:739–52. doi: 10.1016/S2468-1253(20)30077-7, PMID: 32413340

[18] Riley DR Hydes T Hernadez G Zhao SS Alam U Cuthbertson DJ . The synergistic impact of type 2 diabetes and MASLD on cardiovascular, liver, diabetes-related and cancer outcomes. Liver Int. (2024) 44:2538–50. doi: 10.1111/liv.16016, PMID: 38949295

[19] Hagström H Vessby J Ekstedt M Shang Y . 99% of patients with NAFLD meet MASLD criteria and natural history is therefore identical. J Hepatol. (2024) 80:e76–e7. doi: 10.1016/j.jhep.2023.08.026, PMID: 37678723

[20] Zhou XD Kim SU Yip TC Petta S Nakajima A Tsochatzis E . Long-term liver-related outcomes and liver stiffness progression of statin usage in steatotic liver disease. Gut. (2024) 73:1883–92. doi: 10.1136/gutjnl-2024-333074, PMID: 39089860

[21] Chen Z Chen M Zeng P Yang X Li Q . Association of aspirin with all-cause and cardiocerebrovascular mortality in patients with metabolic associated fatty liver disease. Scand J Gastroenterol. (2023) 58:908–14. doi: 10.1080/00365521.2023.2179864, PMID: 36799202

[22] Bambha K Belt P Abraham M Wilson LA Pabst M Ferrell L . Ethnicity and nonalcoholic fatty liver disease. Hepatology. (2012) 55:769–80. doi: 10.1002/hep.24726, PMID: 21987488 PMC3278533

[23] Lim U Ernst T Buchthal SD Latch M Albright CL Wilkens LR . Asian women have greater abdominal and visceral adiposity than Caucasian women with similar body mass index. Nutr Diabetes. (2011) 1:e6. doi: 10.1038/nutd.2011.2, PMID: 23449381 PMC3302135

[24] Maskarinec G Grandinetti A Matsuura G Sharma S Mau M Henderson BE . Diabetes prevalence and body mass index differ by ethnicity: the Multiethnic Cohort. Ethn Dis. (2009) 19:49–55. 19341163 PMC2702477

[25] Mohanty SR Troy TN Huo D O’Brien BL Jensen DM Hart J . Influence of ethnicity on histological differences in non-alcoholic fatty liver disease. J Hepatol. (2009) 50:797–804. doi: 10.1016/j.jhep.2008.11.017, PMID: 19231016

[26] Iwaki M Fujii H Hayashi H Toyoda H Oeda S Hyogo H . Prognosis of biopsy-confirmed metabolic dysfunction- associated steatotic liver disease: A sub-analysis of the CLIONE study. Clin Mol Hepatol. (2024) 30:225–34. doi: 10.3350/cmh.2023.0515, PMID: 38263684 PMC11016478

[27] Kim SK Gignoux CR Wall JD Lum-Jones A Wang H Haiman CA . Population genetic structure and origins of Native Hawaiians in the multiethnic cohort study. PloS One. (2012) 7:e47881. doi: 10.1371/journal.pone.0047881, PMID: 23144833 PMC3492381

[28] Wang J Conti DV Bogumil D Sheng X Noureddin M Wilkens LR . Association of genetic risk score with NAFLD in an ethnically diverse cohort. Hepatol Commun. (2021) 5:1689–703. doi: 10.1002/hep4.1751, PMID: 34558842 PMC8485887

[29] Chang Y Cho YK Kim Y Sung E Ahn J Jung HS . Nonheavy drinking and worsening of noninvasive fibrosis markers in nonalcoholic fatty liver disease: A cohort study. Hepatology. (2019) 69:64–75. doi: 10.1002/hep.30170, PMID: 30019340

[30] Chan TT Chan WK Wong GL Chan AW Nik Mustapha NR Chan SL . Positive hepatitis B core antibody is associated with cirrhosis and hepatocellular carcinoma in nonalcoholic fatty liver disease. Am J Gastroenterol. (2020) 115:867–75. doi: 10.14309/ajg.0000000000000588, PMID: 32149781

[31] Li AA Kim D Kim W Dibba P Wong K Cholankeril G . Disparities in mortality for chronic liver disease among Asian subpopulations in the United States from 2007 to 2016. J Viral Hepat. (2018) 25:1608–16. doi: 10.1111/jvh.12981, PMID: 30112849 PMC6709979

[32] Vijayadeva V Spradling PR Moorman AC Rupp LB Lu M Gordon SC . Hepatitis B virus infection testing and prevalence among Asian and Pacific Islanders. Am J Manag Care. (2014) 20:e98–e104., PMID: 24884958 PMC6542464

[33] Shim CW Park JW Kim SH Kim JS Kim BH Kim SH . Noncirrhotic hepatocellular carcinoma: etiology and occult hepatitis B virus infection in a hepatitis B virus-endemic area. Therap Adv Gastroenterol. (2017) 10:529–36. doi: 10.1177/1756283X17710247, PMID: 28804513 PMC5484439

[34] Vitellius C Desjonqueres E Lequoy M Amaddeo G Fouchard I N’Kontchou G . MASLD-related HCC: Multicenter study comparing patients with and without cirrhosis. JHEP Rep. (2024) 6:101160. doi: 10.1016/j.jhepr.2024.101160, PMID: 39411648 PMC11474187

[35] Weinberg EM Trinh HN Firpi RJ Bhamidimarri KR Klein S Durlam J . Lean americans with nonalcoholic fatty liver disease have lower rates of cirrhosis and comorbid diseases. Clin Gastroenterol Hepatol. (2021) 19:996–1008.e6. doi: 10.1016/j.cgh.2020.06.066, PMID: 32629123

[36] Golabi P Paik J Hwang JP Wang S Lee HM Younossi ZM . Prevalence and outcomes of non-alcoholic fatty liver disease (NAFLD) among Asian American adults in the United States. Liver Int. (2019) 39:748–57. doi: 10.1111/liv.14038, PMID: 30597715

[37] Byrne CD Targher G . NAFLD as a driver of chronic kidney disease. J Hepatol. (2020) 72:785–801. doi: 10.1016/j.jhep.2020.01.013, PMID: 32059982

[38] Xiang J Morgenstern H Li Y Steffick D Bragg-Gresham J Panapasa S . Incidence of ESKD among native hawaiians and pacific islanders living in the 50 US states and pacific island territories. Am J Kidney Dis. (2020) 76:340–9.e1. doi: 10.1053/j.ajkd.2020.01.008, PMID: 32387021

[39] VoPham T Cravero A Feld LD Green P Feng Z Berry K . Associations of race and ethnicity with hepatocellular carcinoma, decompensation, and mortality in US veterans with cirrhosis. Cancer Epidemiol Biomarkers Prev. (2023) 32:1069–78. doi: 10.1158/1055-9965.EPI-22-1291, PMID: 37255388 PMC10390887

[40] Younossi ZM Zelber-Sagi S Lazarus JV Wong VW Yilmaz Y Duseja A . Global consensus recommendations for metabolic dysfunction-associated steatotic liver disease and steatohepatitis. Gastroenterology. (2025) 169:1017–32.e2. doi: 10.1053/j.gastro.2025.02.044, PMID: 40222485

[41] Barba C Manila M Cavalli-Sforza T Cutter J Darnton-Hill I Deurenberg P . Appropriate body-mass index for Asian populations and its implications for policy and intervention strategies. Lancet. (2004) 363:157–63. doi: 10.1016/S0140-6736(03)15268-3, PMID: 14726171

