# Supplementary figures and images for: Ethnic disparities in metabolic dysfunction-associated steatotic liver disease and clinical outcomes
Source: Front Endocrinol (Lausanne). 2026 Jan 9;16:1739137. doi: 10.3389/fendo.2025.1739137 (PMC12827132; doi:10.3389/fendo.2025.1739137)

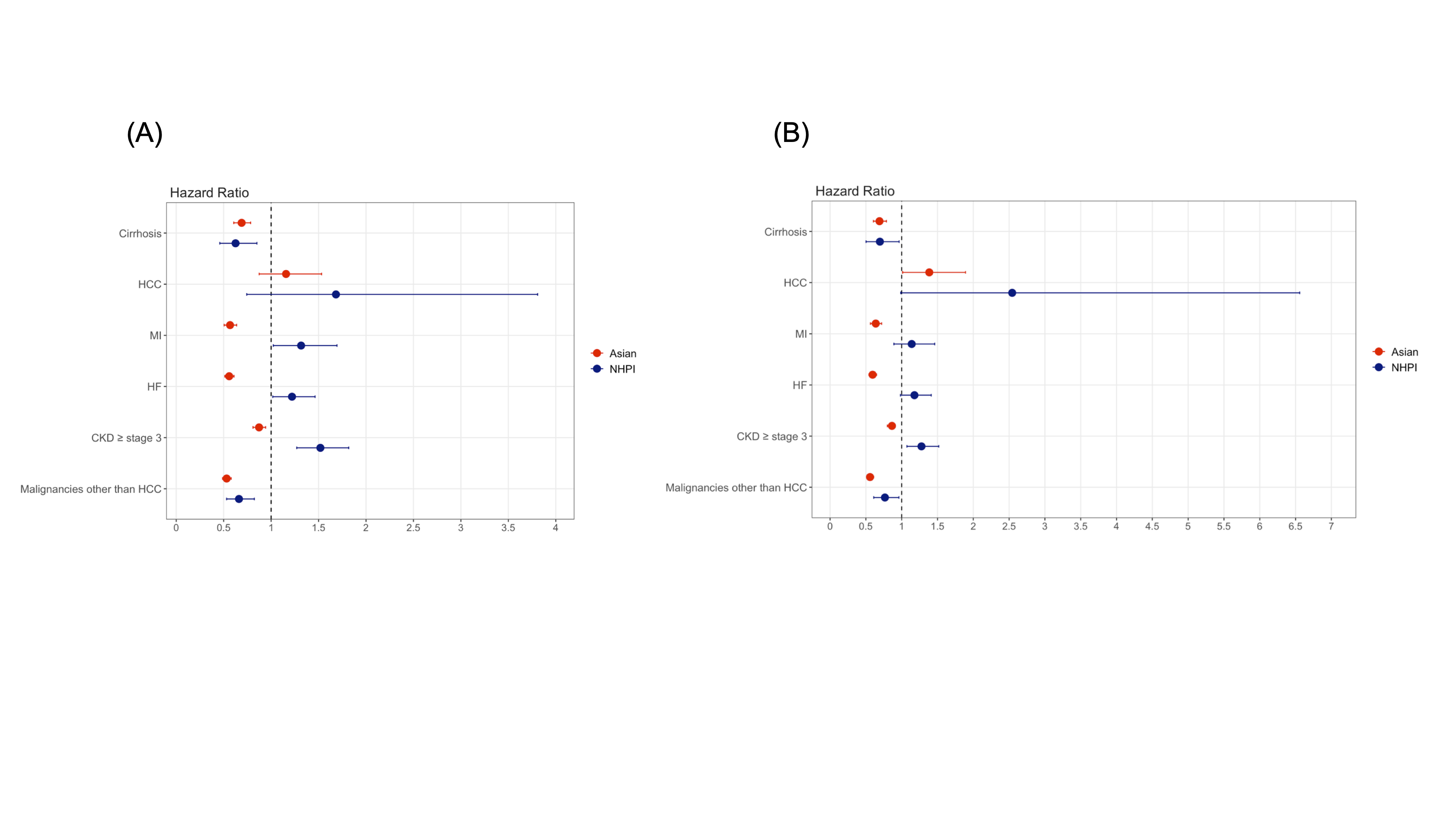

Supplement: Supplementary file 1 [file Image1.tiff]
