# Supplementary material for: Ethnic disparities in metabolic dysfunction-associated steatotic liver disease and clinical outcomes
Source: Front Endocrinol (Lausanne). 2026 Jan 9;16:1739137. doi: 10.3389/fendo.2025.1739137 (PMC12827132; doi:10.3389/fendo.2025.1739137)
Supplement: Supplementary file 2 [file Table1.docx]

**Supplementary Material**

**Supplementary Figure 1 (A) (B)**:

Hazard Ratios and 95% confidence intervals (CI) for clinical outcomes during the follow-up period since the MASLD diagnosis based on the minimum length of follow-up period. (A) minimum follow-up length of 3 years; (B) minimum follow-up length of 5 years.

Asian and NHPI were compared with White after propensity score matching using age, gender, BMI, hypertension, diabetes, hyperlipidemia, and smoking status. Error bars represent 95% CI. Abbreviations:  MASLD, metabolic dysfunction–associated steatotic liver disease; HCC, hepatocellular carcinoma; MI, myocardial infarction; HF, heart failure; CKD, chronic kidney disease; NHPI, Native Hawaiian/Pacific Islander.

**Supplementary Table 1.** List of ICD-10 codes of inclusion criteria of MASLD diagnosis

| Code | Diagnosis |
| --- | --- |
| K76.0 | Fatty liver |
| K75.81 | Nonalcoholic steatohepatitis |
| E11 | Type 2 diabetes mellitus |
| E78.5 | Essential hypertension |
| I10 | Hyperlipidemia |

**Supplementary Table 2.** List of ICD-10 codes of exclusion criteria of MASLD diagnosis

| Code | Diagnosis |
| --- | --- |
| K71 | Toxic liver disease |
| K70 | Alcohol-related liver disease |
| B10-B19 | Viral hepatitis |
| F10 | Alcohol related disorders |
| K75.4 | Autoimmune hepatitis |
| K74.3 | Primary biliary cirrhosis |
| K83.01 | Primary sclerosing cholangitis |
| E83.01 | Wilson’s disease |
| E75.22 | Gaucher disease |
| E83.11 | Hemochromatosis |
| E88.01 | Alpha-1-antitrypsin deficiency |
| I82.0 | Budd-Chiari syndrome |
| K73.0 | Chronic persistent hepatitis |
| K73.2 | Chronic active hepatitis |
| K73.8 | Other chronic hepatitis |
| K73.9 | Chronic hepatitis |
|  |  |

**Supplementary Table 3.** List of ICD-10 codes of clinical outcomes

| **Code** | **Diagnosis** |
| --- | --- |
| K74.6 | Cirrhosis |
| C22.0 | Hepatocellular carcinoma |
| I21 | Myocardial infarction |
| I50 | Heart failure |
|  | Chronic kidney disease |
| M18.3 | Stage 3 (a/b) |
| M18.4 | Stage 4 |
| N18.5 | Stage 5 |
|  | Malignancies other than hepatocellular carcinoma |
| C18 | Colon cancer |
| C19 | Rectosigmoid cancer |
| C20 | Rectum cancer |
| C61 | Prostate cancer |
| C54 | Uterus cancer |
| C50 | Breast cancer |
| C34 | Lung cancer |
| C16 | Stomach cancer |
| C43-44 | Melanoma |
| R99 | All-cause mortality |

**Supplementary Table 4.** List of RxNorm codes of medications

| **RxNorm** | **Medication** |
| --- | --- |
| 1191 | Aspirin |
| 1858285 | Acetylsalicylic acid |
| 83367 | Atorvastatin |
| 41127 | Fluvastatin |
| 6472 | Lovastatin |
| 42463 | Pravastatin |
| 301542 | Rosuvastatin |
| 36567 | Simvastatin |

**Supplementary Table 5.** Baseline characteristics of MASLD patients with ≥ 3-year follow-up before and after propensity-score matching. (A) White versus Asian patients; (B) White versus NHPI patients. Abbreviations: MASLD, metabolic dysfunction–associated steatotic liver disease; NHPI, Native Hawaiian/Pacific Islander.

| **(A) White versus Asian with MASLD** | | | | | | |
| --- | --- | --- | --- | --- | --- | --- |
|  | **Pre-propensity score matching** | |  | **Post-propensity score matching** | |  |
| **Covariate** | **White** | **Asian** | **p-value** | **White** | **Asian** | **p-value** |
| Sample size | 173,700 | 13,622 |  | 13,622 | 13,622 |  |
| Age at index event - mean ± SD (years) | 54.5 ± 13.4 | 53.8 ± 13.7 | < 0.001 | 53.8 ± 13.8 | 53.8 ± 13.7 | 0.762 |
| Sex (%) |  |  |  |  |  |  |
| Female | 56.5 | 55.6 | 0.056 | 56.6 | 55.6 | 0.099 |
| Male | 43.5 | 44.4 | 0.05 | 43.3 | 43.344.4 | 94 |
| Type 2 Diabetes Mellitus | 50,095 (28.8 %) | 4,239 (31.1 %) | < 0.001 | 4,192 (30.8%) | 4,239 (31.1 %) | 0.538 |
| Hyperlipidemia | 68,923 (39.7 %) | 5,823 (42.7 %) | < 0.001 | 5,899 (43.3 %) | 5,823 (42.7 %) | 0.352 |
| Hypertension | 91,326 (52.6 %) | 7,089 (52.0 %) | 0.228 | 7,094 (52.1 %) | 7,089 (52.0 %) | 0.952 |
| BMI - mean ± SD (kg/m^2) | 35.0 ± 7.5 | 29.0 ± 5.4 | < 0.001 | 29.6 ± 5.8 | 29.0 ± 5.4 | < 0.001 |
| BMI group (%) |  |  |  |  |  |  |
| < 25 kg/m^2 | 9.2 | 19.5 | < 0.001 | 19.6 | 19.5 | 0.76 |
| 25 - 30 kg/m^2 | 27.1 | 33.1 | < 0.001 | 34.2 | 33.1 | 0.051 |
| 30 - 40 kg/m^2 | 47.0 | 22.6 | < 0.001 | 23 | 22.6 | 0.427 |
| 40 - 50 kg/m^2 | 18.3 | 3 | < 0.001 | 3.0 | 3.0 | 0.887 |
| 50 < kg/m^2 | 5.1 | 0.7 | < 0.001 | 0.7 | 0.7 | 0.661 |
| Nicotine dependence | 19,254 (11.1%) | 750 (5.5 %) | < 0.001 | 749 (5.5 %) | 750 (5.5 %) | 0.979 |
| Tobacco use | 4,510 (2.6 %) | 151 (1.1 %) | < 0.001 | 135 (1.0 %) | 151 (1.1 %) | 0.342 |

| **(B) White versus NHPI with MASLD** | | | | | | |
| --- | --- | --- | --- | --- | --- | --- |
|  | **Pre-propensity score matching** | |  | **Post-propensity score matching** | |  |
| **Covariate** | **White** | **NHPI** | **p-value** | **White** | **NHPI** | **p-value** |
| Sample size | 173,700 | 2,258 |  | 2,257 | 2,257 |  |
| Age at index event - mean ± SD (years) | 54.5 ± 13.4 | 50.0 ± 13.4 | < 0.001 | 50.0 ± 13.4 | 49.9 ± 13.4 | 0.864 |
| Sex (%) |  |  |  |  |  |  |
| Female | 56.5 | 60.5 | < 0.001 | 60.7 | 60.5 | 0.903 |
| Male | 43.5 | 39.5 | < 0.001 | 39.3 | 39.5 | 0.903 |
| Type 2 Diabetes Mellitus | 50,095 (28.8 %) | 865 (38.3 %) | < 0.001 | 862 (38.2 %) | 864 (38.3 %) | 0.951 |
| Hyperlipidemia | 68,923 (39.7 %) | 1,013 (44.9 %) | < 0.001 | 1,018 (45.1 %) | 1,012 (44.8 %) | 0.858 |
| Hypertension | 91,326 (52.6 %) | 1,357 (60.1%) | < 0.001 | 1,356 (60.1 %) | 1,356 (60.1 %) | 1 |
| BMI - mean ± SD (kg/m^2) | 35.0 ± 7.5 | 36.9 ± 8.7 | < 0.001 | 36.6 ± 8.4 | 36.9 ± 8.7 | 0.368 |
| BMI group (%) |  |  |  |  |  |  |
| < 25 kg/m^2 | 9.2 | 5.4 | < 0.001 | 5.7 | 5.4 | 0.649 |
| 25 - 30 kg/m^2 | 27.1 | 14.2 | < 0.001 | 14.7 | 14.2 | 0.672 |
| 30 - 40 kg/m^2 | 47.0 | 30.1 | < 0.001 | 30 | 30.1 | 0.897 |
| 40 - 50 kg/m^2 | 18.3 | 17.3 | 0.195 | 16.6 | 17.3 | 0.552 |
| 50 < kg/m^2 | 5.1 | 6.2 | 0.014 | 5.6 | 6.2 | 0.376 |
| Nicotine dependence | 19,254 (11.1%) | 359 (15.9 %) | < 0.001 | 335 (14.8 %) | 358 (15.9 %) | 0.342 |
| Tobacco use | 4,510 (2.6 %) | 93 (4.1 %) | < 0.001 | 75 (3.3 %) | 92 (4.1 %) | 0.18 |

**Supplementary Table 6.** Baseline characteristics of MASLD patients with ≥ 5-year follow-up before and after propensity-score matching. (A) White versus Asian patients; (B) White versus NHPI patients. Abbreviations: MASLD, metabolic dysfunction–associated steatotic liver disease; NHPI, Native Hawaiian/Pacific Islander.

| **(A) White versus Asian with MASLD** | | | | | | |
| --- | --- | --- | --- | --- | --- | --- |
|  | **Pre-propensity score matching** | |  | **Post-propensity score matching** | |  |
| **Covariate** | **White** | **Asian** | **p-value** | **White** | **Asian** | **p-value** |
| Sample size | 157,628 | 12,646 |  | 12,646 | 12,646 |  |
| Age at index event - mean ± SD (years) | 54.3 ± 13.3 | 53.8 ± 13.6 | < 0.001 | 53.8 ± 13.7 | 53.8 ± 13.6 | 0.201 |
| Sex (%) |  |  |  |  |  |  |
| Female | 56.7 | 55.9 | 0.085 | 56.1 | 55.9 | 0.800 |
| Male | 43.2 | 44.1 | 0.078 | 44.1 | 43.9 | 0.780 |
| Type 2 Diabetes Mellitus | 45,057 (28.6 %) | 3,926 (31.0 %) | < 0.001 | 3,928 (31.1 %) | 3,926 (31.0 %) | 0.978 |
| Hyperlipidemia | 63,284 (40.1 %) | 5,483 (43.4 %) | < 0.001 | 5,539 (43.8 %) | 5,483 (43.4 %) | 0.478 |
| Hypertension | 83,228 (52.8 %) | 6,663 (52.7 %) | 0.809 | 6,757 (53.4 %) | 6,663 (52.7 %) | 0.236 |
| BMI - mean ± SD (kg/m^2) | 35.0 ± 7.5 | 29.1 ± 5.4 | < 0.001 | 29.8 ± 5.8 | 29.1 ± 5.4 | < 0.001 |
| BMI group (%) |  |  |  |  |  |  |
| < 25 kg/m^2 | 9.1 | 19.3 | < 0.001 | 17.9 | 19.3 | 0.005 |
| 25 - 30 kg/m^2 | 27.2 | 33.2 | < 0.001 | 33.6 | 33.2 | 0.514 |
| 30 - 40 kg/m^2 | 47.3 | 22.8 | < 0.001 | 23.1 | 22.8 | 0.654 |
| 40 - 50 kg/m^2 | 18.4 | 3.0 | < 0.001 | 3.1 | 3.0 | 0.688 |
| 50 < kg/m^2 | 5.0 | 0.7 | < 0.001 | 0.6 | 0.7 | 0.21 |
| Nicotine dependence | 17,255 (10.9 %) | 698 (5.5 %) | < 0.001 | 653 (5.2 %) | 698 (5.5 %) | 0.208 |
| Tobacco use | 4,060 (2.6%) | 140 (1.1 %) | < 0.001 | 90 (0.7 %) | 140 (1.1 %) | 0.001 |

| **(B) White versus NHPI with MASLD** | | | | | | |
| --- | --- | --- | --- | --- | --- | --- |
|  | **Pre-propensity score matching** | |  | **Post-propensity score matching** | |  |
| **Covariate** | **White** | **NHPI** | **p-value** | **White** | **NHPI** | **p-value** |
| Sample size | 157,628 | 2,113 |  | 2,111 | 2,111 |  |
| Age at index event - mean ± SD (years) | 54.3 ± 13.3 | 49.8 ± 13.3 | < 0.001 | 49.9 ± 13.3 | 49.8 ± 13.3 | 0.949 |
| Sex (%) |  |  |  |  |  |  |
| Female | 56.7 | 60.5 | < 0.001 | 60.8 | 60.5 | 0.850 |
| Male | 43.2 | 39.5 | 0.001 | 39.2 | 39.5 | 0.850 |
| Type 2 Diabetes Mellitus | 45,057 (28.6 %) | 810 (38.4 %) | < 0.001 | 777 (36.8 %) | 809 (38.3%) | 0.309 |
| Hyperlipidemia | 63,284 (40.1 %) | 953 (45.1 %) | < 0.001 | 970 (45.9 %) | 952 (45.1 %) | 0.578 |
| Hypertension | 83,228 (52.8 %) | 1,273 (60.3 %) | < 0.001 | 1,268 (60.1 %) | 1,272 (60.3 %) | 0.900 |
| BMI - mean ± SD (kg/m^2) | 35.0 ± 7.5 | 37.0 ± 8.7 | < 0.001 | 36.7 ± 8.4 | 37.0 ± 8.7 | 0.446 |
| BMI group (%) |  |  |  |  |  |  |
| < 25 kg/m^2 | 9.1 | 5.3 | < 0.001 | 5.3 | 5.3 | 0.945 |
| 25 - 30 kg/m^2 | 27.2 | 13.9 | < 0.001 | 14.1 | 13.9 | 0.824 |
| 30 - 40 kg/m^2 | 47.3 | 30.4 | < 0.001 | 29.8 | 30.4 | 0.663 |
| 40 - 50 kg/m^2 | 18.4 | 17.5 | 0.267 | 16.9 | 17.5 | 0.625 |
| 50 < kg/m^2 | 5.0 | 6.3 | 0.009 | 5.4 | 6.3 | 0.213 |
| Nicotine dependence | 17,255 (10.9 %) | 337 (16.0 %) | < 0.001 | 322 (15.3 %) | 336 (15.9 %) | 0.552 |
| Tobacco use | 4,060 (2.6%) | 86 (4.1 %) | < 0.001 | 72 (3.4 %) | 85 (4.0 %) | 0.29 |

**Supplementary Table 7.** Baseline characteristics of MASLD patients without follow-up period limitation before and after propensity-score matching including adjustment with aspirin and statin. (A) White versus Asian patients; (B) White versus NHPI patients.

Abbreviations: MASLD, metabolic dysfunction–associated steatotic liver disease; NHPI, Native Hawaiian/Pacific Islander.

| **(A) White versus Asian with MASLD** | | | | | | |
| --- | --- | --- | --- | --- | --- | --- |
|  | **Pre-propensity score matching** | |  | **Post-propensity score matching** | |  |
| **Covariate** | **White** | **Asian** | **p-value** | **White** | **Asian** | **p-value** |
| Sample size | 559,002 | 40,276 |  | 40,274 | 40,274 |  |
| Age at index event - mean ± SD (years) | 55.7 ± 14.4 | 54.2 ± 15.0 | < 0.001 | 54.0 ± 15.1 | 54.2 ± 15.0 | 0.121 |
| Sex (%) |  |  |  |  |  |  |
| Female | 55.7 | 53.5 | < 0.001 | 53.6 | 53.5 | 0.677 |
| Male | 44.3 | 46.5 | < 0.001 | 46.4 | 46.5 | 0.656 |
| Type 2 Diabetes Mellitus | 160,572 (28.7 %) | 12,729 (31.6 %) | < 0.001 | 12,366 (30.7 %) | 12,727 (31.6 %) | 0.006 |
| Hyperlipidemia | 222,429 (39.8 %) | 16,083 (39.9 %) | 0.575 | 15,984 (39.7 %) | 16,081 (39.9 %) | 0.485 |
| Hypertension | 300,949 (53.8 %) | 20,408 (50.7 %) | < 0.001 | 20,349 (50.5 %) | 20,407 (50.7 %) | 0.683 |
| BMI - mean ± SD (kg/m^2) | 34.7 ± 7.7 | 29.3 ± 5.7 | < 0.001 | 30.0 ± 6.0 | 29.3 ± 5.7 | < 0.001 |
| BMI group (%) |  |  |  |  |  |  |
| < 25 kg/m^2 | 11.9 | 21.9 | < 0.001 | 21.3 | 21.9 | 0.072 |
| 25 - 30 kg/m^2 | 30.5 | 39.1 | < 0.001 | 40.0 | 39.1 | 0.017 |
| 30 - 40 kg/m^2 | 48.5 | 27.2 | < 0.001 | 27.7 | 27.2 | 0.102 |
| 40 - 50 kg/m^2 | 19.1 | 4.1 | < 0.001 | 4.2 | 4.1 | 0.698 |
| 50 < kg/m^2 | 5.4 | 1 | < 0.001 | 0.8 | 1 | 0.012 |
| Nicotine dependence | 73,216 (13.1 %) | 2,498 (6.2 %) | < 0.001 | 2,337 (5.8 %) | 2,498 (6.2 %) | 0.017 |
| Tobacco use | 23,993 (4.3 %) | 678 (1.7 % ) | < 0.001 | 547 (1.4 %) | 678 (1.7 %) | 0.027 |
| Medication |  |  |  |  |  |  |
| Aspirin | 138,562 (24.8%) | 6,528 (16.2%) | < 0.001 | 6,276 (15.6%) | 6,528 (16.2%) | 0.015 |
| Acetylsalicylic acid | 126 (0.02%) | < 10 (--%) | -- | < 10 (--%) | < 10 (--%) | -- |
| Atorvastatin | 112,611 (20.1%) | 7,927 (19.7%) | 0.025 | 7,704 (19.1%) | 7,926 (19.7%) | 0.048 |
| Fluvastatin | 595 (0.1%) | 25 (0.06%) | 0.007 | < 10 (--%) | 25 (0.06%) | 0.011 |
| Lovastatin | 7,032 (1.3%) | 752 (1.9%) | < 0.001 | 573 (1.4%) | 750 (1.9%) | < 0.001 |
| Pravastatin | 29,303 (5.2%) | 1,767 (4.4%) | < 0.001 | 1,619 (4.0%) | 1,767 (4.4%) | 0.009 |
| Rosuvastatin | 43,055 (7.7%) | 2,395 (5.9%) | < 0.001 | 2,150 (5.3%) | 2,395 (5.9%) | < 0.001 |
| Simvastatin | 52,028 (9.3%) | 3,340 (8.3%) | < 0.001 | 3,071 (7.6%) | 3,339 (8.3%) | < 0.001 |

| **(B) White versus NHPI with MASLD** | | | | | | |
| --- | --- | --- | --- | --- | --- | --- |
|  | **Pre-propensity score matching** | |  | **Post-propensity score matching** | |  |
| **Covariate** | **White** | **NHPI** | **p-value** | **White** | **NHPI** | **p-value** |
| Sample size | 559,002 | 5,812 |  | 5,811 | 5,811 |  |
| Age at index event - mean ± SD (years) | 55.7 ± 14.4 | 50.1 ± 14.3 | < 0.001 | 49.9 ± 14.5 | 50.1 ± 14.3 | 0.350 |
| Sex (%) |  |  |  |  |  |  |
| Female | 55.7 | 57.5 | 0.007 | 58.1 | 57.5 | 0.452 |
| Male | 44.3 | 42.5 | 0.007 | 41.8 | 42.5 | 0.430 |
| Type 2 Diabetes Mellitus | 160,572 (28.7 %) | 2,206 (38.0 %) | < 0.001 | 2,168 (37.3 %) | 2,205 (37.9 %) | 0.479 |
| Hyperlipidemia | 222,429 (39.8 %) | 2,417 (41.6 %) | 0.005 | 2,375 (40.9 %) | 2,416 (41.6 %) | 0.44 |
| Hypertension | 300,949 (53.8 %) | 3,346 (57.6 %) | < 0.001 | 3,310 (57.0 %) | 3,345 (57.6 %) | 0.512 |
| BMI - mean ± SD (kg/m^2) | 34.7 ± 7.7 | 36.6 ± 8.9 | < 0.001 | 36.6 ± 8.6 | 36.6 ± 8.8 | 0.883 |
| BMI group (%) |  |  |  |  |  |  |
| < 25 kg/m^2 | 11.9 | 7.3 | < 0.001 | 6.5 | 7.3 | 0.099 |
| 25 - 30 kg/m^2 | 30.5 | 20.2 | < 0.001 | 19.5 | 20.2 | 0.352 |
| 30 - 40 kg/m^2 | 48.5 | 37.6 | < 0.001 | 38.5 | 37.6 | 0.339 |
| 40 - 50 kg/m^2 | 19.1 | 20.6 | 0.004 | 20 | 20.6 | 0.433 |
| 50 < kg/m^2 | 5.4 | 7.5 | < 0.001 | 7.1 | 7.5 | 0.354 |
| Nicotine dependence | 73,216 (13.1 %) | 951 (16.4 %) | < 0.001 | 886 (15.2 %) | 950 (16.3 %) | 0.104 |
| Tobacco use | 23,993 (4.3 %) | 285 (4.9 %) | 0.022 | 241 (4.1 %) | 284 (4.9 %) | 0.055 |
| Medication |  |  |  |  |  |  |
| Aspirin | 138,562 (24.8%) | 1,212 (20.9 %) | < 0.001 | 1,185 (20.4 %) | 1,211 (20.8 %) | 0.551 |
| Acetylsalicylic acid | 126 (0.02%) | 0 (0 %) | 0.252 | < 10 (-- %) | 0 (0 %) | -- |
| Atorvastatin | 112,611 (20.1%) | 1,094 (18.8%) | 0.012 | 1,054 (18.1 %) | 1,094 (18.8 %) | 0.339 |
| Fluvastatin | 595 (0.1%) | < 10 (-- %) | 0.128 | < 10 (-- %) | < 10 (-- %) | -- |
| Lovastatin | 7,032 (1.3%) | 153 (2.6%) | < 0.001 | 119 (2.0 %) | 152 (2.6 %) | 0.043 |
| Pravastatin | 29,303 (5.2%) | 229 (3.9%) | < 0.001 | 209 (3.6 %) | 229 (3.9 %) | 0.330 |
| Rosuvastatin | 43,055 (7.7%) | 172 (3.0%) | < 0.001 | 167 (2.9 %) | 172 (3.0 %) | 0.793 |
| Simvastatin | 52,028 (9.3%) | 500 (8.6%) | 0.066 | 444 (7.6 %) | 499 (8.6 %) | 0.062 |
